# The Quebec Parkinson Network: A Researcher-Patient Matching Platform and Multimodal Biorepository

**DOI:** 10.3233/JPD-191775

**Published:** 2020-01-13

**Authors:** Ziv Gan-Or, Trisha Rao, Etienne Leveille, Clotilde Degroot, Sylvain Chouinard, Francesca Cicchetti, Alain Dagher, Samir Das, Alex Desautels, Janelle Drouin-Ouellet, Thomas Durcan, Jean-François Gagnon, Angela Genge, Jason Karamchandani, Anne-Louise Lafontaine, Sonia Lai Wing Sun, Mélanie Langlois, Martin Levesque, Calvin Melmed, Michel Panisset, Martin Parent, Jean-Baptiste Poline, Ronald B. Postuma, Emmanuelle Pourcher, Guy A. Rouleau, Madeleine Sharp, Oury Monchi, Nicolas Dupré, Edward A. Fon

**Affiliations:** a Department of Neurology and Neurosurgery, McGill University, Montréal, QC, Canada; b Montreal Neurological Institute, McGill University, Montréal, QC, Canada; c Department of Human Genetics, McGill University, Montréal, QC, Canada; d Clinical Research Unit, Montreal Neurological Institute, McGill University, Montréal, QC, Canada; e Faculty of Medicine, McGill University, Montréal, QC, Canada; f Unité des trouves du mouvement André Barbeau, Centre hospitalier de l’Université de Montréal, Montreal, QC, Canada; g Centre de Recherche du CHU de Québec, Axe Neurosciences, Québec, QC, Canada; h Département de Psychiatrie & Neurosciences, Université Laval, Québec, QC, Canada; i McGill Centre for Integrative Neuroscience, Montreal Neurological Institute, Montreal, QC, Canada; j Centre d’Études Avancées en Médecine du Sommeil and Neurology Service, H–pital du Sacré-C–eur de Montréal, Montréal, QC, Canada; k Department of Neurosciences, Université de Montréal, Montréal, QC, Canada; l Faculty of Pharmacy, Université de Montréal, Montreal, Quebec, QC, Canada; m Department of Psychology, Université du Québec à Montréal, Montreal, QC, Canada; n Department of Pathology, Montreal Neurological Institute, McGill University, Montréal, QC, Canada; o Department of Neurology, McGill University Medical Centre, Montréal, QC, Canada; p Division of Neurosciences, CHU de Québec, Université Laval, Québec City, QC, Canada; q Department of Medicine, Faculty of Medicine, Université Laval, Québec City, QC, Canada; r CERVO Brain Research Centre, Québec City, QC, Canada; s Jewish General Hospital, McGill University, Montréal, QC, Canada; t Departments of Clinical Neurosciences and Radiology, University of Calgary, AB, Canada; u Hotchkiss Brain Institute, Cumming School of Medicine, University of Calgary, AB, Canada

**Keywords:** Parkinson disease, Quebec Parkinson Network, registry, biobank

## Abstract

**Background:**

Genetic, biologic and clinical data suggest that Parkinson’s disease (PD) is an umbrella for multiple disorders with clinical and pathological overlap, yet with different underlying mechanisms. To better understand these and to move towards neuroprotective treatment, we have established the Quebec Parkinson Network (QPN), an open-access patient registry, and data and bio-samples repository.

**Objective:**

To present the QPN and to perform preliminary analysis of the QPN data.

**Methods:**

A total of 1,070 consecutively recruited PD patients were included in the analysis. Demographic and clinical data were analyzed, including comparisons between males and females, PD patients with and without RBD, and stratified analyses comparing early and late-onset PD and different age groups.

**Results:**

QPN patients exhibit a male:female ratio of 1.8:1, an average age-at-onset of 58.6 years, an age-at-diagnosis of 60.4 years, and average disease duration of 8.9 years. REM-sleep behavior disorder (RBD) was more common among men, and RBD was associated with other motor and non-motor symptoms including dyskinesia, fluctuations, postural hypotension and hallucinations. Older patients had significantly higher rates of constipation and cognitive impairment, and longer disease duration was associated with higher rates of dyskinesia, fluctuations, freezing of gait, falls, hallucinations and cognitive impairment. Since QPN’s creation, over 60 studies and 30 publications have included patients and data from the QPN.

**Conclusions:**

The QPN cohort displays typical PD demographics and clinical features. These data are open-access upon application (http://rpq-qpn.ca/en/), and will soon include genetic, imaging and bio-samples. We encourage clinicians and researchers to perform studies using these resources.

## INTRODUCTION

Parkinson’s disease (PD) is a common neurodegenerative, age-related movement disorder with a prevalence of 1-2% in individuals older than 60 years of age [1]. As the world’s population ages, the number of PD patients and its burden are projected to dramatically increase in the next decades [2]. The etiology of PD is complex, and includes environmental, genetic and aging-related factors [3]. Furthermore, various genetic, biologic and clinical data suggest that PD is in fact not a single disease, but an umbrella for multiple disorders with clinical and pathological overlap, yet likely with different underlying biological mechanisms. For example, PD patients with *GBA* mutations (*GBA*-PD) and PD patients with *LRRK2* mutations (*LRRK2*-PD) may represent distinct subtypes of PD, in which the typical clinical course and the underlying mechanisms may be different [4–7]. Accordingly, future treatment will likely be directed towards the specific mutations and mechanisms in each of these forms of PD [8]. Similarly, clustering based on clinical variables have demonstrated that clinical subtypes can be distinguished based on baseline symptoms that predict the clinical outcome of PD [9, 10]. A better understanding of this disease and its underlying mechanisms are critical at this point if we wish to identify efficient neuroprotective or neurorestorative treatments.

Recent initiatives, such as the Parkinson’s Progression Marker Initiative (PPMI) and other similar cohorts have emerged [11], to better define and understand PD and its subtypes, identify reliable biomarkers and consequently accelerate therapeutic development. Herein, we present a preliminary analysis of the Quebec Parkinson Network (QPN) patient registry; a longitudinal cohort comprised of individuals with PD recruited in the province of Quebec, Canada. The registry is continuously updated with longitudinal follow-up information and with newly recruited patients. Available information includes demographic data such as age, sex, mother tongue, level of education, smoking status, coffee consumption, as well as clinical data such as presenting and current motor symptoms, non-motor symptoms, Hoehn and Yahr stage, medications. Genetics, imaging and additional clinical measures are currently being collected and will be added soon. This is an open-access cohort, which allows clinicians and researchers, upon application, to access patient data, imaging and samples, in accordance with the open access policy of the Montreal Neurological Institute (MNI) Tanenbaum Open Science Institute (TOSI) [12]. Herein, we present preliminary data analysis from the QPN, and introduce the upcoming Canadian Open Parkinson’s Network (C-OPN), a patient registry inspired by the QPN model that will recruit patients across all Canadian provinces.

## METHODS

### Population and data collection

At the time of collecting the data for this paper (January 2019), a total of 1,070 PD patients were enrolled into the QPN and analysis is presented on this cohort of 1,070 patients. However, enrolment is ongoing, with a total of >1,400 patients recruited by August 2019 at the time of completing the first draft of this manuscript. All patients were diagnosed by a movement disorder specialist in the province of Quebec according to the MDS criteria or previously published criteria for patients who were recruited before the publication of the MDS criteria [13]. We included here all the patients, i.e., clinically established and clinically probable based on the MDS criteria, and PD patients diagnosed using previous criteria such as the UK Brain Bank criteria. The majority of patients (76.9%) were recruited at the MNI, the Centre Hospitalier de l’Université de Montréal (CHUM), and the Centre Hospitalier Universitaire de Québec (CHUQ). Data were collected by using the QPN Questionnaire (Supplementary Material), which is completed for each participant with the help of a neurologist or a trained research assistant. This questionnaire collects data on demographics, diagnosis, motor symptoms, treatment and medications, lifestyle and environmental risk factors, magnetic resonance imaging (MRI) compatibility, sleeping habits, family history of PD or PD-related disorders, non-motor symptoms and comorbidities, as well as additional tests such as cognitive and psychiatric evaluations (Table 1). More details on the type of tests used can be found in the Supplementary Material. The QPN categorizes the collected data according to criteria commonly used for PD clinical trials. Longitudinal clinical data are being collected from consenting patients every 18 months. Since most of the patients were recruited in the last 18 months, longitudinal data are currently available for a small portion of the patients. While not analysed in the current study, these data will be available for future studies.

**Table 1:** Demographic and clinical variables collected in the Quebec Parkinson Network

Demographic data	Clinical data
•Sex	•Date of symptom onset
•Date of birth	•Date of diagnosis
•Laterality	•Confirmation of diagnosis
•First language, spoken language, number of languages spoken	•Atypical Parkinsonian syndromes (MSA, PSP, CBD, DLB, FTD, ET)
•Residence (house, institution, other)	•Modified Hoehn and Yahr stage^a^
•Situation (single, in relationship, other caregiver)	•Asymmetry of symptoms
•Education	•First and dominant symptoms (tremor, rigidity, bradykinesia, postural instability)
•Smoking history	•Presence of dyskinesia, motor fluctuations, motor block
•Alcohol and coffee consumption	•History of fall
•Occupation and home location (urban, rural)	•Anti-PD treatment history
•Environmental exposure to neurotoxins	•RBD and sleep disorder
•Frequency of head trauma	•Comorbidities (hypertension, hypotension, diabetes, constipation)
•MRI compatibility	•Presence and treatment of psychiatric disorders (anxiety, depression, bipolar disorder, hallucinations, impulsive/compulsive behaviour)
•Family history of PD and PD-related disease	•Cognitive impairment
	•Presence of dementia

CBD, corticobasal degeneration; DLB, dementia with Lewy bodies; ET, essential tremor; FTD, frontotemporal dementia; MSA, multiple system atrophy; PD, Parkinson’s disease; PSP, progressive supranuclear palsy; RBD, REM (rapid eye movement) sleep behaviour disorder. ^a^Stages are described in Supplementary Table 1.

**Fig.1:**
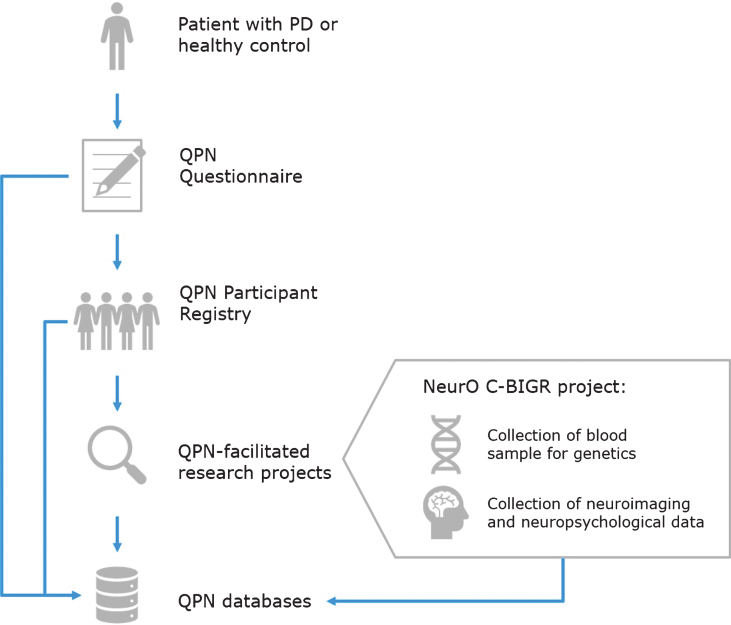
Quebec Parkinson Network (QPN) workflow for collection of patient data. Patients with PD or healthy controls who are recruited to the QPN Participant Registry must complete the QPN Questionnaire. Active members of the QPN Participant Registry may be selected for participation in QPN-facilitated research projects, such as the NeurO C-BIGR project which involves collection of blood samples for genetics and iPSCs derivation, as well as neuroimaging and neuropsychological data. Patient data collected through the QPN Questionnaire, medical records of patients in the QPN Participant Registry, and obtained via QPN-facilitated research projects are entered into the QPN databases.

Patients recruited through the QPN Participant Registry are automatically given the option to be included in the Neuro Open Science Clinical Biologic Imaging and Genetic Repository (NeurO C-BIGR), a multi-modal bio-repository of the MNI (https://www.mcgill.ca/c-bigneuro/). The workflow of this process is depicted in Fig. 1. The objective of this ongoing project is to collect blood from all participants in the registry for genotyping/sequencing and for the generation of patient-specific induced pluripotent stem cell (iPSC) lines. In addition, during the first phase of the QPN, 200 of these participants will also undergo magnetoencephalography (MEG), MRI, a neuropsychological evaluation, a motor evaluation, and provide a recorded speech sample.

All patients recruited through the QPN have signed an informed consent form at enrollment, and the study protocol was approved by the institutional research ethics board.

### Statistical analysis

Data are presented as percentages for categorical variables or as averages±standard deviation (SD) for continuous variables. To compare frequencies of categorical variables, either *χ*^2^ test or Fisher Exact test were used. If adjustment for covariates was required, binary logistic regression models were used. To compare continuous variables, T-test or analysis of variance (ANOVA) were used, and when adjustment for covariates was required, we used linear regression models.

Sub-group and stratified analyses were performed to examine different correlations between sub-groups and PD demographic and clinical characteristics. These included sex-stratified analysis (including 691 males and 379 females), PD patients with and without probable rapid eye movement (REM) sleep behavior disorder (RBD), determined by the validated RBD1Q [14] (PD+RBD, *n* = 381, PD-RBD, *n* = 570, respectively), stratification by age groups (0–50 years, *n* = 45, 51–60 years, *n* = 171, 61–70 years, *n* = 371, 71–80 years, *n* = 362, and >80 years, *n* = 107), disease duration (0–5 years, *n* = 194), 6–10 years, *n* = 163, and >10 years, *n* = 159) and early-onset PD (EOPD, defined as age at onset [AAO] under 50 years, *n* = 108) vs late-onset PD (LOPD, 50 years or above, *n* = 410). Bonferroni correction for multiple comparisons was performed as required. All statistical analyses were performed using SPSS 23.0 (IBM Inc.).

## RESULTS

### Demographic and clinical characteristics of patients in the Quebec Parkinson Network

**Fig.2:**
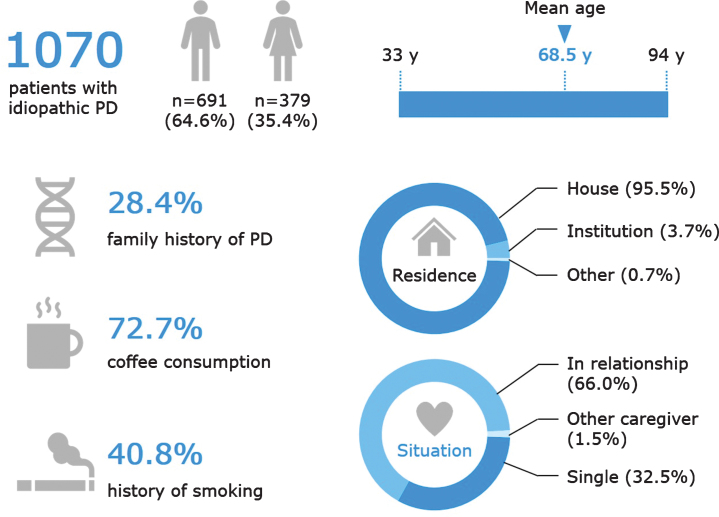
Key demographic characteristics of patients in the Quebec Parkinson Network cohort. Data are presented for the 1070 patients with PD, except the following for which data were not available for all patients: age (*n* = 1056) and coffee consumption (*n* = 985). Family history of PD refers to having a mother, father, sibling, child, or other relation (uncle, aunt, cousin, niece, or nephew) with PD or PD-related disorder. Coffee consumption refers to current coffee drinkers and does not include former coffee drinkers. A history of smoking refers to current and former smokers.

**Fig.3:**
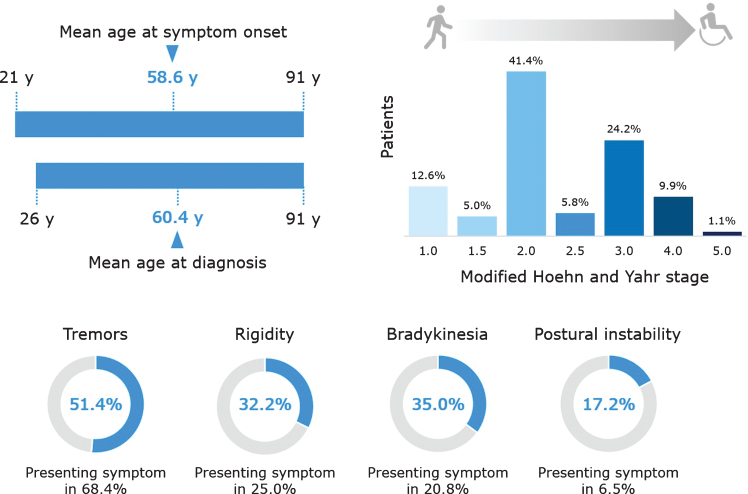
Key clinical characteristics of patients in the Quebec Parkinson Network cohort. Data are presented for the 1070 patients with PD, except the following for which data were not available for all patients: age at symptom onset (*n* = 518), age at diagnosis (*n* = 921), modified Hoehn and Yahr stage (*n* = 637), postural instability (*n* = 697), tremor as presenting symptom (*n* = 863), rigidity as presenting symptom (*n* = 748), bradykinesia as presenting symptom (*n* = 787), and postural instability as presenting symptom (*n* = 724).

Figures 2 and 3 depict the main demographic and clinical characteristics of the first 1,070 PD patients from the QPN Participant Registry. Supplementary Tables 2 and 3 include more detailed data on the main demographic and clinical variables. This cohort is typical of other PD tertiary and academic centers’ patient cohorts, with a male:female ratio of about 1.8:1, average AAO of 58.6±11.3 years (available data for *n* = 518), age at diagnosis of 60.4±11.0 years (available data for *n* = 921) and average current age of 68.5±9.8 years (available data for *n* = 1056). The average disease duration is 8.9±6.8 years (available data for *n* = 516), with an average Hoehn & Yahr score of 2.35±0.88 (available data for *n* = 637). Of the 518 patients for whom data on AAO was available, 108 (20.9%) had EOPD. Supplementary Table 3 details other motor and non-motor clinical characteristics of the QPN cohort, such as probable RBD (reported in 40.1% of patients with available data, *n* = 951), self-reported dyskinesia (47.2%, available data for *n* = 835) and freezing of gait (16.2%, available data for *n* = 1070). Non-motor symptoms are particularly prevalent, with self-reported anxiety being the most common (56.9%, available data for *n* = 865).

### Sex differences in the clinical presentation of PD

To examine whether specific clinical characteristics were associated with sex, we performed a sex-stratified analysis comparing male (*n* = 691) and female (*n* = 379) patients with PD (Table 2). There were no statistically significant differences in age, AAO, age at diagnosis, time to diagnosis, and Hoehn & Yahr stage between male and female patients. Female patients had a longer disease duration than males, with nominal significance (9.71±7.58 years vs 8.43±6.23 years, respectively, *p* = 0.047). The difference was not statistically significant after correction for multiple comparisons or after adjustment for age of onset, which was younger in females, although not statistically significant (57.45±12.03 years vs 59.24±10.73 years, respectively, *p* = 0.08).

**Table 2:** Sex stratified analysis of PD symptoms in the Quebec Parkinson Network

Symptom	Patients (%)		*P*-value^a^
	Male *n* = 691	Female *n* = 379
Presenting symptoms
Tremor	339/691 (49.1)	211/379 (55.7)	0.038^b^
Rigidity	225/691 (32.6)	120/379 (31.7)	0.763^b^
Bradykinesia	247/691 (35.7)	128/379 (33.8)	0.518^b^
Postural instability	72/439 (16.4)	48/257 (18.7)	0.443^b^
Asymmetry	451/691 (65.3)	280/379 (73.9)	0.004^b^
Other symptoms during disease course
Dyskinesia	223/515 (43.3)	171/320 (53.4)	0.004^c^
Fluctuations	206/489 (42.1)	129/296 (43.6)	0.690^b^
Freezing of gait	109/691 (15.80)	64/379 (16.9)	0.636^b^
Falls	80/691 (11.6)	57/379 (15.0)	0.105^b^
Postural hypotension	36/506 (7.1)	34/283 (12.0)	0.020^b^
Constipation	140/509 (27.5)	89/291 (30.6)	0.354^b^
Anxiety	292/547 (53.4)	200/318 (62.9)	0.006^b^
Depression	216/531 (40.7)	140/308 (45.5)	0.177^b^
Hallucinations	110/518 (21.2)	65/296 (22.0)	0.809^b^
Impulsive or compulsive behavior	4/488 (0.8)	5/283 (1.8)	0.238 ^b^
Other psychiatric disorders	5/485 (1.0)	1/276 (0.4)	0.316^b^
Cognitive impairment	183/564 (32.4)	102/313 (32.6)	0.966^b^
Dementia	15/429 (3.5)	8/255 (3.1)	0.801^b^
Sleep disorders	254/488 (52.0)	146/284 (51.4)	0.864^b^
RBD	269/608 (44.2)	112/343 (32.7)	<0.001^b^^b^.

RBD, Rapid eye movement (REM) sleep behavior disorder. ^a^Bonferroni correction for multiple comparisons set the cut-off *p* value for statistical significance on 0.0025. ^b^Not significant after adjustment for disease duration. ^c^Significant after adjustment for age and disease duration.

When compared to male patients, tremor, postural hypotension, anxiety, dyskinesia and asymmetry of symptoms were more prevalent in females (Table 2). None of these associations were statistically significant after correction for multiple comparisons (Bonferroni-corrected *p* value of 0.0025) or after adjustment for disease duration, except for dyskinesia which remained statistically significant after age and/or disease duration adjustment, but not after Bonferroni correction. The only association that remained statistically significant after both Bonferroni correction and adjustment for age and/or disease duration was probable RBD, which was more common in males (44.2% vs 32.7%, *p* < 0.001). There were no statistically significant differences in the prevalence of presenting symptoms (symptoms reported by the patients at the onset of the diseases) between male and female patients.

### Comparison between PD with and without probable RBD

Since it was previously reported that RBD may represent specific subtypes of PD [15], we compared PD+RBD (*n* = 381) patients to PD-RBD (*n* = 570) patients. Table 3 details the results of the analysis. After Bonferroni correction for multiple comparisons (set the cut-of *p* value on 0.0028) and after adjustment for sex and disease duration, higher frequencies of dyskinesia, fluctuations, postural hypotension and hallucinations were associated with PD+RBD (Table 3). Constipation remained nominally more common in PD+RBD after correction for sex and disease duration but not statistically significant after Bonferroni correction.

**Table 3:** Analysis of symptoms stratified by RBD status

Symptom	Patients (%)		*P*-value^a^
	Non-RBD *n* = 570	RBD *n* = 381
Presenting symptoms
Tremor	323/570 (56.7)	201/381 (52.8)	<0.235^c^
Rigidity	187/570 (32.8)	141/381 (37.0)	<0.182^c^
Bradykinesia	201/570 (35.3)	153/381 (40.2)	<0.126^c^
Postural instability	67/426 (15.7)	49/256 (19.1)	<0.251^c^
Asymmetry	425/570 (74.6)	270/381 (70.9)	<0.208^c^
Other symptoms during disease course
Dyskinesia	193/481 (40.1)	180/321 (56.1)	<0.001^c^
Fluctuations	167/461 (36.2)	156/301 (51.8)	<0.001^c^
Freezing of gait	98/570 (17.2)	70/381 (18.4)	<0.640^c^
Falls	67/570 (11.8)	64/381 (16.8)	<0.027^b^
Postural hypotension	31/479 (6.5)	37/286 (12.9)	<0.002^c^
Constipation	121/482 (25.1)	102/294 (34.7)	<0.004^c^
Anxiety	279/507 (55.0)	191/326 (58.6)	<0.312^c^
Depression	190/491 (38.7)	149/315 (47.3)	<0.016^b^
Hallucinations	82/483 (17.0)	87/302 (28.8)	<0.001^c^
Impulsive or compulsive behavior	3/464 (0.6)	6/285 (2.1)	<0.075^c^
Other psychiatric disorders	2/460 (0.4)	4/280 (1.4)	<0.144^c^
Cognitive impairment	162/463 (35.0)	114/305 (37.4)	<0.500^c^
Dementia	12/418 (2.9)	9/251 (3.6)	<0.608^c^

RBD, Rapid eye movement (REM) sleep behavior disorder. ^a^Bonferroni correction for multiple comparisons set the cut-off *p* value for statistical significance on 0.0028. ^b^Not significant after adjustment for disease duration. ^c^Significant after adjustment for sex and disease duration.

### Analysis of symptoms by age groups and disease duration

To examine whether specific symptoms are associated with age groups and disease duration, we performed the following analyses: a) Comparison between EOPD (*n* = 108) and LOPD (*n* = 410, Table 4); b) Comparison by current age groups (see Methods for details, Supplementary Table 4); and c) Comparison by disease duration (<5 years, *n* = 194; 6–10 years, *n* = 163;>10 years, *n* = 159, Supplementary Table 5).

**Table 4:** Comparison of symptoms between early- and late-onset PD

Symptom	Patients (%)		*P*-value^a^
	Late onset *n* = 410	Early onset *n* = 108
Presenting symptoms
Tremor	269/410 (65.6)	65/108 (60.2)	<0.295^b^
Rigidity	143/410 (34.9)	46/108 (42.6)	<0.138^b^
Bradykinesia	146/410 (35.6)	38/108 (35.2)	<0.935^b^
Postural instability	90/362 (24.9)	15/100 (15.0)	<0.037^c^
Asymmetry	302/410 (73.7)	80/108 (74.1)	<0.930^b^
Other symptoms during disease course
Dyskinesia	119/367 (32.4)	55/102 (53.9)	<0.001^b^
Fluctuations	111/359 (30.9)	46/98 (46.9)	<0.003^b^
Freezing of gait	86/410 (21.0)	28/108 (25.9)	<0.269^b^
Falls	62/410 (15.1)	25/108 (23.1)	<0.047^b^
Postural hypotension	34/395 (8.6)	11/104 (10.6)	<0.533^b^
Constipation	139/392 (35.5)	32/104 (30.8)	<0.371^b^
Anxiety	206/402 (51.2)	57/106 (53.8)	<0.643^b^
Depression	150/401 (37.4)	42/105 (40.0)	<0.626^b^
Hallucinations	61/398 (15.3)	22/105 (21.0)	<0.167^b^
Impulsive or compulsive behavior	2/387 (0.5)	0/102 (0.0)	<0.467^b^
Other psychiatric disorders	1/382 (0.3)	1/102 (1.0)	<0.315^b^
Cognitive impairment	112/338 (33.1)	25/84 (29.8)	<0.554^b^
Dementia	5/335 (1.5)	1/93 (1.1)	<0.762^b^
Sleep disorders	184/392 (46.9)	57/105 (54.3)	<0.181^b^
RBD	141/395 (35.7)	38/105 (36.2)	<0.925^b^

RBD, Rapid eye movement (REM) sleep behavior disorder. ^a^Bonferroni correction for multiple comparisons set the cut-off *p* value for statistical significance on 0.0026. ^b^Not significant after adjustment for disease duration. ^c^Significant after adjustment for age.

Patients with EOPD had a higher rate of dyskinesia compared to LOPD (53.9% vs 32.4%, respectively, *p* < 0.001, statistically significant after Bonferroni correction for multiple comparisons), as well as fluctuations (46.9% vs 30.9%, *p* = 0.003, not statistically significant after Bonferroni correction) and falls (23.1% vs 15.1%, *p* = 0.047, not statistically significant after Bonferroni correction). The frequency of postural instability was higher among LOPD compared to EOPD (24.9% and 15.0%, respectively, *p* = 0.037, not statistically significant after Bonferroni correction). As expected, age of patients was associated with higher Hoehn & Yahr stage (*p* < 0.001), even after adjustment for AAO, disease duration and sex. Furthermore, older patients had significantly higher rates of constipation (*p* < 0.001, Supplementary Table 4), and cognitive impairment (*p* < 0.001), which remained statistically significant after adjustment for disease duration and after Bonferroni correction for multiple comparisons. Longer disease duration was associated with higher rates of dyskinesia (*p* < 0.001, Supplementary Table 5), fluctuations (*p* = 0.001), freezing of gait (*p* < 0.001), falls (*p* < 0.001), hallucinations (*p* < 0.001) and cognitive impairment (*p* < 0.001), all statistically significant after adjustment for age, age at onset and sex, and after correction for multiple comparisons.

## DISCUSSION

### The Quebec Parkinson Network cohort as an open-access, representative PD cohort

Our preliminary analyses demonstrate that the QPN cohort is a typical, representative PD cohort from tertiary and academic centers, comparable to previous reports describing different clinical and epidemiological characteristics and correlations within similar PD cohorts. For example, the QPN cohort’s epidemiological characteristics such as sex distribution, age at onset, diagnosis and enrollment are in line with previously published cohorts [16–18]. The associations between different clinical variables (e.g., disease duration and cognitive decline [19, 20], RBD and hallucinations [21, 22], etc.) further exemplify that this cohort is typical for academic centers. Of note, since now web-based recruitment to QPN is available, almost one-third of QPN patients are from community-based clinics. However, availability of web access and knowledge may also somewhat bias the recruitment. Another limitation is that data collected by questionnaire and from patients’ medical files (when accessible) may be less accurate.

The QPN (http://rpq-qpn.ca/en/) collects data and samples through 4 main channels: the QPN Questionnaire (see Supplementary Material), medical records of patients enrolled in the QPN Participant Registry, research projects facilitated by the QPN (described later), and the NeurO C-BIGR (see Methods). In addition, other data are being collected for all patients, or in some cases for subgroups of patients, such as genetic, imaging, neuropsychological and neuropsychiatric data etc. Genetic data are being collected from all patients for whom DNA is available (currently >800 patients, out of >1,200 patients that have been enrolled by the completion of this paper), including genotyping using a custom single nucleotide polymorphism (SNP) array with >700,000 SNPs (Backbone of the OmniExpress array with custom content of the NeuroX SNP-chop, Illumina, Inc.), and targeted next generation sequencing of ∼50 PD-related genes, including *GBA*, *LRRK2*, *SNCA, MAPT* and others [23–25]. For a large number of patients (currently  400), peripheral blood mononuclear cells (PBMCs) are being collected, and iPSCs and neuronal models are being generated by the MNI-iPSC platform (https://mniopenresearch.org/articles/3-1) for specific studies as required. These data and bio-samples are being made available by applying for specific research projects through the QPN website and obtaining approval from the QPN scientific committee and the tissue and data committee of the Neuro C-BIGR. The success of our model may encourage funding of similar initiative, preferably in under-represented parts of the world where clinical and genetic data on PD patients are less available.

### Initiating studies with the Quebec Parkinson Network data and samples

The QPN has three main research axes: 1) Clinical and Treatment Research, 2) Non-Motor Symptoms, and 3) Molecular and Cellular Biology. Since its creation in June 2013, the QPN has supported more than 60 research projects involving participants from the registry, leading to more than 30 peer-reviewed publications to date. QPN-facilitated research projects are initiated when an investigator requests to use the registry, to access existing data or to enroll QPN patients for their study and collect additional data or samples for their study. The QPN selects patients from the registry for participation in the study based on the inclusion and exclusion criteria provided by the investigator. If the investigator approves inclusion of the participant in their study, they may contact the participant. An initial contact form, indicating whether or not the participant will be included in the study, is submitted by the investigator to the QPN and this information is documented by the QPN for each participant. Participants who are not included in the study remain eligible to be selected for other studies. Participants may be involved in several studies simultaneously; however, the QPN ensures that participants already taking part in a study are not selected for another study deemed incompatible with the first. In this way, the registry provides access to patients for investigators conducting research in PD. As the QPN Participant Registry continues to grow, the QPN is consolidating data collected through the questionnaire, patient medical records, and QPN-facilitated research studies, such as NeurO C-BIGR, in a centralized, open-access database (LORIS [Longitudinal Online Research and Imaging System]) [26–28]. All epidemiological, clinical, neuroimaging, neuropsychological, and biological data will then be made publicly accessible to promote data sharing throughout the PD research community, ultimately leading to new research projects and analyses. Applications for data and for new studies can be made through the QPN website (http://rpq-qpn.ca/en/).

### Recent studies and trials facilitated by the Quebec Parkinson Network

A number of recently published studies using QPN patients in the Clinical and Treatment Research axis have investigated wearable movement detection technology that would allow neurologists to monitor patients beyond the clinic. Data collected from inertial measurement units (IMUs) worn by patients with PD were used to assess motor symptoms including tremor, bradykinesia, and freezing of gait, as well as drug-induced dyskinesia [29]. Using this inertial sensor technology, algorithms were developed that can detect and segment movements in patients performing a Timed Up and Go task [30] or carrying out activities of daily living in a simulated living space [31]. IMU data were also used to identify a turning signature that discriminates between older-age adults and older-age patients with PD, as well as between patients in ON and OFF medication states [32]. The algorithms are currently being tested in a smartphone app that will be used to detect and measure tremor and bradykinesia in a QPN-supported project.

Of importance to advancing treatment for patients with PD and PD-related disorders, the QPN has assisted with participant recruitment for 2 ongoing clinical trials. The MOVES-PD trial is a phase II global study to assess the dynamics, efficacy, and safety of GZ/SAR402671 (Genzyme/Sanofi), a small molecule inhibitor of glucosylceramide synthase [33], in patients with early-stage PD who carry a mutation in the gene encoding glucocerebrosidase (*GBA*) (NCT02906020). A second trial is assessing the efficacy, safety, tolerability, and pharmacokinetics of ABBV-8E12 (AbbVie), a humanized anti-Tau antibody [34], in patients with progressive supranuclear palsy (PSP), an atypical parkinsonian syndrome (NCT02985879).

The second QPN research axis focuses on the non-motor symptoms of PD through the use of neuroimaging and neuropsychological evaluations. Several published studies have identified potential predictors of specific non-motor symptoms, including depressive symptoms, cognitive impairment, and dementia. The severity of depressive symptoms in non-demented patients with PD was shown to be associated with higher dosages of levodopa [35] and with higher rates of cortical thinning [36]. Cognitive deficits in patients with PD were correlated with RBD [37], a longer history of hypertension and higher pulse pressure [38], and a history of smoking [39]. A study of dementia in patients with PD identified age, male sex, baseline RBD, orthostatic hypotension, and mild cognitive impairment (MCI) as significant predictors of dementia, and the co-existence of RBD, MCI, and orthostatic hypotension at baseline was found to be the strongest determinant for the development of dementia [40]. A recent study suggested that transcranial magnetic stimulation may improve cognition in PD patients [41]. Ongoing projects in this research axis are continuing to investigate cognitive function in patients with PD, as well as auditory, speech, and olfactory functions.

The third QPN research axis is Molecular and Cellular Biology. The majority of publications from this axis are studies on the genetics of PD. Targeted next generation sequencing of candidate genes in patients with PD and healthy controls has supported roles for specific genetic variants in the pathogenesis of the disease, such as variants in *GCH1*, *MAPT* [23], and *SMPD1* [43], and demonstrated lack of association with other genes [44–46]. Furthermore, recruiting patients from the QPN Participant Registry provides an opportunity to access a large number of patients who are of French-Canadian ancestry, which was critical for the recent identification of a founder mutation in the *GBA* gene in French-Canadian patients with PD or RBD [25]. Genome-wide association study (GWAS) data from QPN patients have also been used for recent large-scale genetic analyses of mitochondria-related genes and GWAS of AAO in PD [47, 48]. The GWAS data are currently being used to accurately determine the ancestry of all participants, and these data will be made available for the users of QPN data. Ongoing projects in this axis aim to identify novel genetic determinants of PD and PD-related disorders, and to study genetic models in dopaminergic neurons and midbrain organoids derived from QPN PD patients.

### Future directions and the Canadian Open Parkinson’s Network

The QPN’s success in building a robust patient registry with associated epidemiological and clinical data and in establishing standardized processes for facilitating research projects has served as the model for the creation of a Canadian national PD network. The recently funded Canadian Open Parkinson Network (C-OPN) will involve 8 major movement disorder centres across 4 Canadian provinces: the Hotchkiss Brain Institute and Movement Disorders program at the University of Calgary; the McGill Parkinson Program at the MNI and Montreal General Hospital (McGill University); L’Unité des Troubles du Mouvement André Barbeau at the Université de Montréal; La Clinique des Troubles du Mouvement du Centre Hospitalier Universitaire de Québec at the CHU de Québec-Université Laval; the Parkinson Research Consortium (University of Ottawa); the Movement Disorders Centre at Toronto Western Hospital (University of Toronto); the Movement Disorders Program at the University of Alberta; and the Pacific Parkinson’s Research Centre at the University of British Columbia. C-OPN aims to create an open-access panel of patient-derived PD stem cell lines with associated clinical and imaging data, in partnership with the Open Science iPSC Platform and C-BIGR at the MNI. Similarly to QPN, C-OPN has 3 major research initiatives. First, C-OPN will establish a national registry of patients with PD and PD-related disorders containing comprehensive longitudinal clinical information to facilitate patient recruitment for large-scale projects or clinical trials. Second, C-OPN will set up an anonymized database for neuropsychological information, imaging data, and second-generation wearable device data. Third, C-OPN will store biological samples in an open biorepository from which genetic and cellular samples and data will be processed, curated, and made available to the scientific community in Canada and beyond. The goals of C-OPN are to find new technological platforms for imaging and wearable data, identify biomarkers and platforms for drug discovery, and ultimately develop novel stratification methods based on multidimensional data to better predict disease trajectory and inform treatment strategies for PD.

## CONFLICT OF INTEREST

ZGO reports consultation fees from Lysosomal Therapeutics Inc. (LTI), Idorsia, Denali, Prevail Therapeutics, Inception Sciences (Ventus), all outside the submitted work. AD received research grants from Flamel Ireland Limited, Pfizer, Canopy Growth, Biron as well as honoraria from speaking engagements from UCB and Biogen. None of the financial disclosures is relevant to the submitted work. RBP reports personal fees from Takeda, Roche/Prothena, Teva Neurosciences, Novartis Canada, Biogen, Boehringer Ingelheim, Theranexus, GE HealthCare, Jazz Pharmaceuticals, Abbvie, Jannsen, and Otsuko, all outside the submitted work.

All other authors have no conflict of interests to report.

## Supplementary Material

Supplementary Tables
