# Supplementary material for: The Quebec Parkinson Network: A Researcher-Patient Matching Platform and Multimodal Biorepository
Source: J Parkinsons Dis. 2020 Jan 13;10(1):301–13. doi: 10.3233/JPD-191775 (PMC7029361; doi:10.3233/JPD-191775)
Supplement: Supplementary Tables [file jpd-10-jpd191775-s001.docx]

Supplementary Table 1. Modified Hoehn and Yahr stages.

| **Stage** | **Description** |
| --- | --- |
| 1.0 | Unilateral involvement only |
| 1.5 | Unilateral and axial involvement |
| 2.0 | Bilateral involvement without impairment of balance |
| 2.5 | Mild bilateral disease with recovery on pull test |
| 3.0 | Mild to moderate bilateral disease; some postural instability; physically independent |
| 4.0 | Severe disability; still able to walk or stand unassisted |
| 5.0 | Wheelchair bound or bedridden unless aided |

**Supplementary Table 2. Demographic characteristics available in the Quebec Parkinson’s Network. Data are updated for October 2018.**

| **Characteristic** | **Patients (n/N [%])** |
| --- | --- |
| **Age at time of analysis (N=1056)** |  |
| Mean ± SD (years) | 68.5 ± 9.81 (range: 33–94) |
| **Sex** |  |
| Men | 691/1070 (64.6) |
| Women | 379/1070 (35.4) |
| **Laterality** |  |
| Right | 968/1070 (90.5) |
| Left | 102/1070 (9.5) |
| **First language** |  |
| French | 760/1045 (72.7) |
| English | 129/1045 (12.3) |
| Other^*^ | 156/1045 (14.9) |
| **Spoken language** |  |
| French | 890/1070 (83.2) |
| English | 480/1070 (44.9) |
| **Number of languages spoken** |  |
| Average ± SD (years) | 1.5 ± 0.75 (range: 1–6) |
| **Residence** |  |
| House | 1022/1070 (95.5) |
| Institution | 40/1070 (3.7) |
| Other | 8/1070 (0.7) |
| **Situation** |  |
| Single | 348/1070 (32.5) |
| In relationship | 706/1070 (66.0) |
| Other caregiver | 16/1070 (1.5) |
| **Education** |  |
| Primary | 200/1070 (18.7) |
| Secondary | 266/1070 (24.9) |
| CÉGEP | 190/1070 (17.8) |
| University | 414/1070 (38.7) |
| **Smoking** |  |
| Yes | 42/1070 (3.9) |
| No | 633/1070 (59.2) |
| Formerly | 395/1070 (36.9) |
| **Coffee drinker** |  |
| Yes | 716/985 (72.7) |
| No | 187/985 (19.0) |
| Formerly | 82/985 (8.3) |
| **Occupation location** |  |
| Urban | 777/878 (88.5) |
| Rural | 101/878 (11.5) |
| **Home location** |  |
| Urban | 685/880 (77.8) |
| Rural | 195/880 (22.2) |
| **Environmental exposure to neurotoxins** |  |
| Yes | 54/611 (8.8) |
| No | 557/611 (91.2) |
| **Frequency of head trauma** |  |
| Never | 867/1070 (81.0) |
| Once only | 144/1070 (13.5) |
| A few times | 55/1070 (5.1) |
| Often | 4/1070 (0.4) |
| **MRI compatible** |  |
| Yes | 886/1070 (82.8) |
| No | 184/1070 (17.2) |
| **Family history of PD or PD-related disease** |  |
| Any | 304/1070 (28.4) |
| Father | 62/1070 (5.8) |
| Mother | 45/1070 (4.2) |
| Sibling | 57/1070 (5.3) |
| Child | 2/1070 (0.2) |
| Other relation^†^ | 138/1070 (12.9) |
| None | 766/1070 (71.6) |
| **Genetics available** |  |
| Yes | 644/997 (64.6) |
| No | 353/997 (35.4) |
| **Imaging data available** |  |
| Yes | 13/680 (1.9) |
| No | 667/680 (98.1) |
| CÉGEP, Collège d’enseignement général et professionnel, a step between secondary school and university in Quebec; ND, not determined; PD, Parkinson’s disease; SD, standard deviation.  ^*^Other mother tongues included: African (dialect), Afrikaans, Arabic, Armenian, Chinese (simplified), Chinese (traditional), Czech, Danish, Dutch, Finnish, German, Greek, Haitian Creole, Hebrew, Hindi, Hungarian, Irish, Italian, Lithuanian, Persian, Polish, Portuguese, Romanian, Russian, Spanish, Ukrainian, Urdu, Vietnamese, and Yiddish.  ^†^Other relations included: uncle, aunt, cousin, nephew, and niece. | |

**Supplementary Table 3. Main clinical characteristics of the Quebec Parkinson’s Network. Data are updated for October 2018.**

| **Characteristic** | **Patients (n/N [%])** |
| --- | --- |
| **Age at symptom onset (N=518)** |  |
| Mean ± SD (years) | 58.6 ± 11.26 (range: 21–91) |
| **Age at diagnosis (N=921)** |  |
| Mean ± SD (years) | 60.4 ± 10.96 (range: 26–91) |
| **Tremor as presenting symptom** |  |
| Yes | 590/863 (68.4) |
| No | 273/863 (31.6) |
| **Rigidity as presenting symptom** |  |
| Yes | 187/748 (25.0) |
| No | 561/748 (75.0) |
| **Bradykinesia as presenting symptom** |  |
| Yes | 164/787 (20.8) |
| No | 623/787 (79.2) |
| **Postural instability as presenting symptom** |  |
| Yes | 47/724 (6.5) |
| No | 677/724 (93.5) |
| **Modified Hoehn and Yahr stage** |  |
| 1.0 | 80/637 (12.6) |
| 1.5 | 32/637 (5.0) |
| 2.0 | 264/637 (41.4) |
| 2.5 | 37/637 (5.8) |
| 3.0 | 154/637 (24.2) |
| 4.0 | 63/637 (9.9) |
| 5.0 | 7/637 (1.1) |
| Mean ± SD | 2.35 ± 0.88 |
| **Symptoms at recruitment** |  |
| **Tremors** |  |
| Yes | 550/1070 (51.4) |
| No | 520/1070 (48.6) |
| **Rigidity** |  |
| Yes | 345/1070 (32.2) |
| No | 725/1070 (67.8) |
| **Bradykinesia** |  |
| Yes | 375/1070 (35.0) |
| No | 695/1070 (65.0) |
| **Postural instability** |  |
| Yes | 120/696 (17.2) |
| No | 576/696 (82.8) |
| **Asymmetry of symptoms** |  |
| Any | 731/1070 (68.3) |
| Right | 385/731 (52.7) |
| Left | 346/731 (47.3) |
| **Dyskinesia** |  |
| Yes | 394/835 (47.2) |
| No | 441/835 (52.8) |
| **Motor fluctuations** |  |
| Yes | 335/785 (42.7) |
| No | 450/785 (57.3) |
| **Freezing of gait** |  |
| Yes | 173/1070 (16.2) |
| No | 897/1070 (83.8) |
| **Falls** |  |
| Yes | 137/1070 (12.8) |
| No | 933/1070 (87.2) |
| **Anti-PD treatment^a^** |  |
| Levodopa | 511/697 (73.3) |
| Dopaminergic agonist | 14/697 (2.0) |
| Levodopa + dopaminergic agonist | 149/697 (21.4) |
| Other | 12/697 (1.7) |
| None | 11/697 (1.6) |
| ND, not determined; PD, Parkinson’s disease; SD, standard deviation.  ^a^ Data on other medications for PD is currently collected and is not available for the current manuscript. | |

**Supplementary Table 4. Age-stratified analysis of PD patients in the QPN.**

| **Symptom** | **Patients (%)** | | | | | | | | ***p*-value^a^** | | |  |
| --- | --- | --- | --- | --- | --- | --- | --- | --- | --- | --- | --- | --- |
|  | **Age 0-50** | **Age 51-60** | **Age 61-70** | **Age 71-80** | | **Age >80** | |  | | |  |  |
|  | **n = 45** | **n = 171** | **n = 371** | **n = 362** | | **n = 107** | |  |  |  |  |  |
| **Tremor** | 24/45 (53.3) | 87/171 (50.9) | 198/371 (53.4) | 188/362 (51.9) | | 51/107 (47.7) | | 0.878 | | |  |  |
| **Rigidity** | 17/45 (37.8) | 61/171 (35.7) | 130/371 (35.0) | 98/362 (27.1) | | 37/107 (34.6) | | 0.111 | | |  |  |
| **Bradykinesia** | 13/45 (28.9) | 63/171 (36.8) | 121/371 (32.6) | 133/362 (36.7) | | 44/107 (41.1) | | 0.397 | | |  |  |
| **Postural instability** | 3/31 (9.7) | 14/113 (12.4) | 37/252 (14.7) | 52/230 (22.6) | | 14/67 (20.9) | | **0.050^c^** | | |  |  |
| **Asymmetry** | 36/45 (80.0) | 121/171 (70.8) | 269/371 (72.5) | 237/362 (65.5) | | 66/107 (61.7) | | **0.045^b^** | | |  |  |
| **Dyskinesia** | 13/33 (39.4) | 64/132 (48.5) | 156/308 (50.6) | 132/281 (47.0) | | 28/78 (35.9) | | 0.173 | | |  |  |
| **Fluctuations** | 11/29 (37.9) | 62/127 (48.8) | 119/290 (41.0) | 115/266 (43.2) | | 28/71 (39.4) | | 0.578 | | |  |  |
| **Freezing of gait** | 2/45 (4.4 | 21/171 (12.3) | 65/371 (17.5) | 69/362 (19.1) | | 16/107 (15.0) | | 0.057 | | |  |  |
| **Falls** | 2/45 (4.4) | 9/171 (5.3) | 58/371 (15.6) | 56/362 (15.5) | | 11/107 (10.3) | | **0.002^c^** | | |  |  |
| **Postural hypotension** | 5/34 (14.7) | 10/125 (8.0) | 29/284 (10.2) | 24/267 (9.0) | | 2/76 (2.6) | | 0.213 | | |  |  |
| **Constipation** | 0/34 (0.0) | 33/126 (26.2) | 77/290 (26.6) | 100/271 (36.9) | | 18/76 (23.7) | | **<0.001^c^** | | |  |  |
| **Anxiety** | 24/37 (64.9) | 79/137 (57.7) | 173/312 (55.4) | 169/292 (57.9) | | 47/84 (56.0) | | 0.845 | | |  |  |
| **Depression** | 15/37 (40.5) | 71/135 (52.6) | 124/302 (41.1) | 119/284 (41.9) | | 27/78 (34.6) | | 0.093 | | |  |  |
| **Hallucinations** | 6/36 (16.7) | 20/131 (15.3) | 58/292 (19.9) | 72/275 (26.2) | | 18/77 (23.4) | | 0.102 | | |  |  |
| **Impulsive or compulsive behavior** | 0/35 (0.0) | 4/121 (3.3) | 2/281 (0.7) | 3/256 (1.2) | | 0/75 (0.0) | | 0.160 | | |  |  |
| **Other psychiatric disorders** | 0/35 (0.0) | 1/121 (0.8) | 2/273 (0.7) | 3/254 (1.2) | | 0/75 (0.0) | | 0.847 | | |  |  |
| **Cognitive impairment** | 4/37 (10.8) | 29/137 (21.2) | 100/308 (32.5) | 116/298 (38.9) | | 35/83 (42.2) | | **<0.001^c^** | | |  |  |
| **Dementia** | 0/32 (0.0) | 2/106 (1.9) | 8/252 (3.2) | 8/222 (3.6) | | 5/69 (7.2) | | 0.280 | | |  |  |
| **Sleep disorders** | 16/33 (48.5) | 79/125 (63.2) | 139/280 (49.6) | 126/253 (49.8) | | 39/78 (50.0) | | 0.104 | | |  |  |
| **RBD** | 9/41 (22.0) | 60/152 (39.5) | 147/336 (43.8) | 132/322 (41.0) | | 33/97 (34.0) | | 0.059 | | |  |  |
| RBD, rapid eye movement (REM) sleep behavior disorder | | | | |  | |  | | |  | | |
| ^a^ Bonferrony correction for multiple comparisons set the cut-off p value for statistical significance on 0.0025 | | | | | | | | | |  | | |
| ^b^ Not significant when adjusted for disease duration | | | | | | | | | | | | |
| ^c^ Significant when adjusted for disease duration | | | | | | | | | | | | |

**Supplementary Table 5. Analysis of PD patients in the QPN by disease duration.**

| **Symptom** | **Patients (%)** | | | | | | **p-value^a^** |  |
| --- | --- | --- | --- | --- | --- | --- | --- | --- |
|  | **Disease duration (years)** | | | | | |  |  |
|  | **0-5** | **6-10** | | **>10** | | |  |  |
|  | **n = 194** | **n = 163** | | **n = 159** | | |  |  |
| **Tremor** | 131/194 (67.5) | 111/163 (68.1) | | 91/159 (57.2) | | | 0.068 |  |
| **Rigidity** | 66/194 (34.0) | 65/163 (39.9) | | 57/159 (35.8) | | | 0.51 |  |
| **Bradykinesia** | 63/194 (32.5) | 59/163 (36.2) | | 61/159 (38.4) | | | 0.501 |  |
| **Postural instability** | 43/172 (25.0) | 28/145 (19.3) | | 34/143 (23.8) | | | 0.460 |  |
| **Asymmetry** | 153/194 (78.9) | 118/163 (72.4) | | 110/159 (69.2) | | | 0.105 |  |
| **Dyskinesia** | 27/162 (16.7) | 54/151 (35.8) | | 93/154 (60.4) | | | <0.001^c^ |  |
| **Fluctuations** | 44/158 (27.8) | 45/151 (29.8) | | 68/146 (46.6) | | | 0.001^c^ |  |
| **Freezing of gait** | 26/194 (13.4) | 35/163 (21.5) | | 53/159 (33.3) | | | <0.001^c^ |  |
| **Falls** | 17/194 (8.8) | 24/163 (14.7) | | 45/159 (28.3) | | | <0.001^c^ |  |
| **Postural hypotension** | 14/186 (7.5) | 16/156 (10.3) | | 15/155 (9.7) | | | 0.646 |  |
| **Constipation** | 59/187 (31.6) | 53/153 (34.6) | | 58/154 (37.7) | | | 0.496 |  |
| **Anxiety** | 92/190 (48.4) | 86/159 (54.1) | | 85/157 (54.1) | | | 0.463 |  |
| **Depression** | 73/189 (38.6) | 53/159 (33.3) | | 66/156 (42.3) | | | 0.256 |  |
| **Hallucinations** | 11/188 (5.9) | 30/158 (19.0) | | 41/155 (26.5) | | | <0.001^c^ |  |
| **Impulsive or compulsive behavior** | 1/182 (0.5) | 0/153 (0.0) | | 1/152 (0.7) | | | 0.624 |  |
| **Other psychiatric disorders** | 0/178 (0.0) | 1/152 (0.7) | | 1/152 (0.7) | | | 0.555 |  |
| **Cognitive impairment** | 25/157 (15.9) | 47/131 (35.9) | | 64/132 (48.5) | | | <0.001^c^ |  |
| **Dementia** | 0/150 (0.0) | 3/140 (2.1) | | 3/136 (2.2) | | | 0.191 |  |
| **Sleep disorders** | 89/185 (48.1) | 82/154 (53.2) | | 70/156 (44.9) | | | 0.33 |  |
| **RBD** | 60/184 (32.6) | 50/157 (31.8) | | 69/157 (43.9) | | | 0.041^b^ |  |
| RBD, rapid eye movement (REM) sleep behavior disorder | | | | | | |  |  |
| ^a^ Bonferrony correction for multiple comparisons set the cut-off p value for statistical significance on 0.0025 | | | | | | | | |
| ^b^ Not significant when adjusted for age of onset | | |  | |  | |  |  |
| ^c^ Significant when adjusted for age, age of onset, and sex | | | | | |  |  |  |
|  |  |  | |  | | |  |  |
